# 568. Evaluation of the VITEK 2 XL Antimicrobial Susceptibility Testing for Non-fermenter Gram-Negative Bacilli: The Importance of Continuing to Monitor AST Systems

**DOI:** 10.1093/ofid/ofad500.637

**Published:** 2023-11-27

**Authors:** Cecilia G Carvalhaes, Paul Rhomberg, Nathan Veeder, Nabina Gurung, Mariana Castanheira

**Affiliations:** JMI Laboratories, North Liberty, IA; JMI Laboratories, North Liberty, IA; JMI Laboratories, North Liberty, IA; JMI Laboratories, North Liberty, IA; JMI Laboratories, North Liberty, IA

## Abstract

**Background:**

*Pseudomonas aeruginosa* (PSA), *Acinetobacter calcoaceticus-baumannii* complex (ACB), and *Stenotrophomonas maltophilia* (SM) are difficult-to-treat pathogens contributing significantly to mortality. Monitoring the accuracy of automated susceptibility (S) systems like VITEK 2 against contemporaneous clinical isolates is crucial. This study compared VITEK2 to the broth microdilution method (BMD) when reporting S results against a challenge set of NF-GNB isolates.

**Figure:**
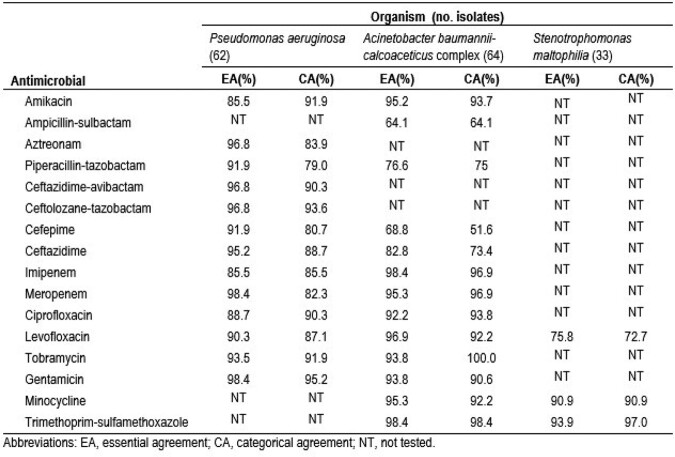

**Methods:**

A challenge set of PSA (62 isolates; 21 carbapenem-resistant [CR] and 20 cefepime [CEP]-R), ACB (64; 21-CR and 21 CEP-R), and SM (33; 11 trimethoprim-sulfamethoxazole [TS]-R) were collected (1/patient) in 2019–2021 from 43 medical centers located in 9 US Census Divisions and identified by MALDI-TOF MS. Isolates were S tested by VITEK 2 (AST-N802+XN15 cards) and CLSI BMD. A total of 1,737 isolate/antimicrobial (ATM) combinations were evaluated for essential (EA) and categorical (CA) agreements and error rates. FDA-approved VITEK 2 breakpoints were applied to both methods to mimic the real-world practice.

**Results:**

Overall, VITEK 2 EA/CA rates were 90.6%/87.0%. Additionally, EA/CA rates were 93.1%/87.8%, 88.6%/86.3%, and 86.9%/86.9% for PSA, ACB, and SM, respectively. The EA/CA rates for each ATM and organism combination are displayed in the Table. All ATMs showed > 90% EA against PSA, except amikacin (AMK), imipenem, and ciprofloxacin (CIP). However, CA rates >90% were noted for AMK, ceftazidime-avibactam, ceftolozane-tazobactam, CIP, tobramycin, and gentamycin against PSA. VITEK 2 CA rates for all other ATMs tested against PSA ranged from 79.0% to 88.7%. All ATMs but ampicillin-sulbactam, piperacillin-tazobactam, CEF, and ceftazidime exhibited EA and CA rates > 90% against ACB. Minocycline and TS showed > 90% EA and CA rates against SM, but rates were lower for levofloxacin (EA/CA, 75.8%/72.7%).

**Conclusion:**

The collection of NF-GNB pathogens tested in this study have been designated serious threats, so accurate AST results are critical for diagnostic stewardship. VITEK 2 EA/CA rates were > 90% for most of ATMs tested against this challenging collection of NF-GNB; however, β-lactam agents showed CA rates < 88% against PSA (80% of discordances were minor errors).

**Disclosures:**

**Cecilia G. Carvalhaes, MD, PhD**, AbbVie: Grant/Research Support|bioMerieux: Grant/Research Support|Cipla: Grant/Research Support|CorMedix: Grant/Research Support|Melinta: Grant/Research Support|Pfizer: Grant/Research Support **Paul Rhomberg, BS, MT(ASCP)**, bioMerieux: Grant/Research Support|Melinta: Grant/Research Support|Pfizer: Grant/Research Support **Nathan Veeder, AA**, bioMerieux: Grant/Research Support **Nabina Gurung, n/a**, bioMerieux: Grant/Research Support **Mariana Castanheira, PhD**, AbbVie: Grant/Research Support|Basilea: Grant/Research Support|bioMerieux: Grant/Research Support|Cipla: Grant/Research Support|CorMedix: Grant/Research Support|Entasis: Grant/Research Support|Melinta: Grant/Research Support|Paratek: Grant/Research Support|Pfizer: Grant/Research Support|Shionogi: Grant/Research Support

